# Editorial: Bio-Pathological Markers in the Diagnosis and Therapy of Cancer

**DOI:** 10.3390/cancers15051484

**Published:** 2023-02-26

**Authors:** Giuseppe Broggi, Lucia Salvatorelli

**Affiliations:** Department of Medical and Surgical Sciences and Advanced Technologies “G.F. Ingrassia”, Anatomic Pathology, University of Catania, 95123 Catania, Italy

**Keywords:** cancer, diagnosis, prognosis, biomarkers, therapy

## Introduction

Identifying novel biomarkers with diagnostic, prognostic and predictive value in terms of therapeutic response is a current topic in the clinical practice of oncologists, pathologists and medical researchers in general [1]. The introduction of molecular techniques capable of investigating the genetic landscape of human neoplasms provides further impetus to this need [1]. The present collection included thirteen original research (OR) articles, eight review papers and one study protocol, in which various aspects of the topic of the Special Issue have been investigated.

Youn et al. investigated the differential transcriptomic landscapes of pediatric and adult chronic myeloid leukemia (CML) cells [2]. Through the use of RNA sequencing, they found that several genes were differentially up- and/or down-regulated in pediatric CML CD34-positive cells to those observed in both pediatric unaffected CD34-positive cells and adult CML CD34-positives cells [2]. Many of these genes were involved in the Rho pathway, whose altered regulation could explain some clinical differences between the adult and the pediatric form of CML [2]. The radiological study by Hsu et al. aimed to identify some thin-slide Computed Tomography (CT) features that could reliably predict lung invasive adenocarcinoma (IA) among its radiological mimickers presenting as pure ground-glass nodules (pGGNs), including atypical adenomatous hyperplasia (AAH), adenocarcinoma in situ (AIS) and minimally invasive adenocarcinoma (MIA) [3]. As pGGNs smaller than 2 cm are relatively difficult to biopsy adequately and lung IA exhibits a poorer prognosis than its mimickers, in this clinical context, it is crucial to identify reliable radiological features that can direct clinicians to a decision on whether to perform a biopsy or not [3]. On a series of 181 pGGNs smaller than 2 cm, the authors found that the following factors were associated with an increased risk of IA: (i) larger size, (ii) lobulation and (iii) air cavity; in addition, the multivariate analysis showed that the latter was a statistically significant predictor of IA [3]. Gassenmaier et al. performed a clinico-pathologic and immunohistochemical study to investigate the differential expression of PReferentially expressed Antigen in Melanoma (PRAME) between thin melanomas (Breslow thickness: ≤1.0 mm) and dysplastic compound nevi (SDN) and its prognostic significance in the former [4]. In more detail, diffuse PRAME staining was shown to strongly favor the diagnosis of melanoma over SDN, while no significant differences in PRAME expression were observed between metastasizing and non-metastasizing thin melanomas [4]. Berrino et al. reported the prevalence rates of gene fusions in human cancer types [5]; 125 specimens from patients affected by different malignancies, including colorectal and lung cancer and melanoma, were analyzed using RNA-based targeted next-generation sequencing [5]. They found higher fusion rates in their cohort than those reported in Memorial Sloane Kettering Cancer Centre (MSKCC) cohorts, emphasizing the need for more frequent application of these techniques in clinical practice [5]. Hoyer et al. aimed to compare the sensitivity and sensibility of individualized viral-cellular-junction test (vcj-PCR) + cytology with those of standard methods (high-risk HPV-DNA test + cytology) in the post-treatment follow-up of women affected by high-risk squamous intraepithelial lesion/cervical intraepithelial neoplasm grade 3 (HSIL/CIN3) [6]. The former technique, in spite of its high specificity, was found to be less sensitive indetecting recurrent CIN2/3 than the latter [6]. In the research by Schwertner et al.,Nectin-1 expression was demonstrated to be a significant predictor of susceptibility of malignant melanoma cells to Oncolytic Herpes Simplex Virus both in vitro and in vivo [7]. Caja et al. found on Dextran sulphate sodium (DSS)-induced colitis and azoxymethane (AOM)-induced colorectal carcinogenesis rat models a significant downregulation of TGF-β1 and inflammatory cytokines, along with a collagen scaffold remodeling, in both diseases, indicating that the latter could be considered as a potential preneoplastic feature of colonic mucosa [8]. Serine and arginine-rich splicing factor 1 (SRSF1) is a splicing factor protein whose expression and function have been recently found to be altered in several human malignancies [9]; our research group investigated the potential diagnostic role of this protein in neuropathologist’s practice, demonstrating its frequent immunohistochemical expression among adult diffuse astrocytomas and oligodendrogliomas, along with its negativity among ependymomas and pilocytic astrocytomas [10]. Similarly, the immunohistochemical study by Piombino et al. showed that Wilms’ tumor 1 (WT1) immunoreactivity could represent a strong diagnostic tool to distinguish dermatofibrosarcoma protuberans from other dermal/subcutaneous bland-looking mesenchymal spindle cell lesions, such as dermatofibromas, deep fibrous histiocytomas, neurofibromas, spindle cell lipomas, dermal scars, nodular fasciitis, skin leiomyomas and solitary fibrous tumors [11]. Engels et al. investigated the sensibility and specificity rates of the detection of lymph node metastases in prostate cancer by one-step nucleic acid amplification (OSNA) [12]; when compared with “conventional” histopathologic examination of lymph nodes, high levels of concordance with this method were seen [12]. According to Chen et al., the loss of Tid1/DNAJA3 Co-Chaperone was found to stimulate tumor growth and recurrence risk in surgically resected hepatocellular carcinoma [13]. Mayer and colleagues demonstrated a strong correlation between restricted water diffusion in diffusion-weighted magnetic resonance imaging (DW-MRI) and tumor hypoxia, with the overexpression of B-lymphocyte induced maturation protein (Blimp-1) and vascular endothelial growth factor (VEGF), as surrogate markers for hypoxia, on tissue specimens of the matched patients [14]. The last OR article by Puglisi et al. focused on the grade of sensitivity to radiotherapy of cancer stem cells (CSCs)isolated from locally advanced rectal cancer biopsies [15]; based on these findings, the authors hypothesized that an in vitro prediction model of the potential response to radiotherapy could be done to personalize the treatment of these patients and to avoid radiation toxicity [15].

Among the review paper, the first one by Paydary et al. studied the literature evidence related to the use of immune-checkpoint inhibitors (ICIs) in gastro-esophageal cancer therapy, emphasizing that first-line ICI may mostly be used in patients exhibiting high PD-L1 levels, irrespectively of histopathology or anatomic location [16]. The potential role of cardiac biomarkers such as troponin and N-terminal prohormone of brain natriuretic peptide as less expensive and useful tools to predict, early diagnose and monitor different cancer-related cardiac conditions, was reviewed by Semeraro et al., who recognized the limitations reported in the literature, that often did not allow for a secure and standardized use of these markers in clinical practice [17]. Similarly, Honrubia-Peris and colleagues provided readers with an update on the available biomarkers capable of predicting therapeutic response to ICIs in advanced non-small cell lung cancer (NSCLC) [18]; some future perspectives, including the potential use of non-invasive markers (liquid biopsies or plasma determinations) were also discussed [18]. Despite being the most common intraocular malignancy in adults, uveal melanoma (UM) is a relatively unusual tumor, and its rarity is reflected in the paucity of currently known valid prognostic and predictive factors [19,20]. Gajdzis et al. filled the need to provide the scientific community with “the status of the art” concerning prognostic and immunohistochemical markers of this lesion, emphasizing that some of these could be useful in predicting the metastatic risk of UM patients [21]. The role of oncometabolites and their relationship with cancer initiation and progression were narrated and discussed by Beyoğlu et al. [22], while González-Gascón-y-Marín and colleagues performed a critical review of the predictive biomarkers of chronic lymphocytic leukemia (CLL), including 11q deletion, *TP53* alterations and *IGHV* and *NOTCH1* mutations [23]. The research group by Torrisi et al. summarized the literature data related to the biological impact of hypoxia on glioblastoma (GBM) invasiveness and acquisition of radio-resistant phenotype with activation of SRC proto-oncogene non-receptor tyrosine kinase, providing some suggestions to potentially overcome the current limitations in GBM treatment [24]. Russo et al. compared the previously reported rates of sensitivity and specificity and the oncologic outcomes of different techniques such as photodynamic diagnosis (PDD) fluorescence, narrow-band imaging (NBI) and white light cystoscopy (WLC) in visualizing and sampling non-muscle invasive bladder cancer NMIBC [25]; from their data meta-analysis, tumor resection with PDD and NBI was shown to exhibit lower recurrence rates and higher diagnostic sensitivity than WLC alone, and NBI resulted in better disease sensitivity and specificity than conventional WLC [25]. Finally, Diaz-del Castillo and colleagues presented a study protocol aimed at investigating the type, location and intensity of pain, its consequences on the quality of life of multiple myeloma (MM) patients, as well as the potential damage suffered by bone nerves in this condition [26].

We thank all the authors of the above-mentioned papers and all the reviewers who have dedicated their time and efforts to the success of this Special Issue.

## References

[1] Broggi G., Salvatorelli L. (2020). Bio-Pathological Markers in the Diagnosis and Therapy of Cancer. Cancers.

[2] Youn M., Smith S.M., Lee A.G., Chae H.-D., Spiteri E., Erdmann J., Galperin I., Jones L.M., Donato M., Abidi P. (2021). Comparison of the Transcriptomic Signatures in Pediatric and Adult CML. Cancers.

[3] Hsu W.-C., Huang P.-C., Pan K.-T., Chuang W.-Y., Wu C.-Y., Wong H.-F., Yang C.-T., Wan Y.-L. (2021). Predictors of Invasive Adenocarcinomas among Pure Ground-Glass Nodules Less Than 2 cm in Diameter. Cancers.

[4] Gassenmaier M., Hahn M., Metzler G., Bauer J., Yazdi A.S., Keim U., Garbe C., Wagner N.B., Forchhammer S. (2021). Diffuse PRAME Expression Is Highly Specific for Thin Melanomas in the Distinction from Severely Dysplastic Nevi but Does Not Distinguish Metastasizing from Non-Metastasizing Thin Melanomas. Cancers.

[5] Berrino E., Bragoni A., Annaratone L., Fenocchio E., Carnevale-Schianca F., Garetto L., Aglietta M., Sarotto I., Casorzo L., Venesio T. (2021). Pursuit of Gene Fusions in Daily Practice: Evidence from Real-World Data in Wild-Type and Microsatellite Instable Patients. Cancers.

[6] Hoyer H., Mehlhorn G., Scheungraber C., Hagemann I., Hirchenhain C., Woelber L., Stolte C., Hampl M., Scherbring S., Denecke A. (2021). Evaluation of Integrated HPV DNA as Individualized Biomarkers for the Detection of Recurrent CIN2/3 during Post-Treatment Surveillance. Cancers.

[7] Schwertner B., Lindner G., Toledo Stauner C., Klapproth E., Magnus C., Rohrhofer A., Gross S., Schuler-Thurner B., Öttl V., Feichtgruber N. (2021). Nectin-1 Expression Correlates with the Susceptibility of Malignant Melanoma to Oncolytic Herpes Simplex Virus In Vitro and In Vivo. Cancers.

[8] Čaja F., Stakheev D., Chernyavskiy O., Kubinová L., Křížan J., Dvořák J., Rossmann P., Štěpánková R., Makovický P., Makovický P. (2021). Local Immune Changes in Early Stages of Inflammation and Carcinogenesis Correlate with the Collagen Scaffold Changes of the Colon Mucosa. Cancers.

[9] Broggi G., Lo Giudice A., Di Mauro M., Asmundo M.G., Pricoco E., Piombino E., Caltabiano R., Morgia G., Russo G.I. (2021). SRSF-1 and microvessel density immunohistochemical analysis by semi-automated tissue microarray in prostate cancer patients with diabetes (DIAMOND study). Prostate.

[10] Broggi G., Salvatorelli L., Barbagallo D., Certo F., Altieri R., Tirrò E., Massimino M., Vigneri P., Guadagno E., Maugeri G. (2021). Diagnostic Utility of the Immunohistochemical Expression of Serine and Arginine Rich Splicing Factor 1 (SRSF1) in the Differential Diagnosis of Adult Gliomas. Cancers.

[11] Piombino E., Broggi G., Barbareschi M., Castorina S., Parenti R., Bartoloni G., Salvatorelli L., Magro G. (2021). Wilms’ Tumor 1 (WT1): A Novel Immunomarker of Dermatofibrosarcoma Protuberans—An Immunohistochemical Study on a Series of 114 Cases of Bland-Looking Mesenchymal Spindle Cell Lesions of the Dermis/Subcutaneous Tissues. Cancers.

[12] Engels S., Brautmeier L., Reinhardt L., Wasylow C., Hasselmann F., Henke R.P., Wawroschek F., Winter A. (2021). Evaluation of Fast Molecular Detection of Lymph Node Metastases in Prostate Cancer Patients Using One-Step Nucleic Acid Amplification (OSNA). Cancers.

[13] Chen K.-Y., Huang Y.-H., Teo W.-H., Chang C.-W., Chen Y.-S., Yeh Y.-C., Lee C.-J., Lo J.-F. (2021). Loss of Tid1/DNAJA3 Co-Chaperone Promotes Progression and Recurrence of Hepatocellular Carcinoma after Surgical Resection: A Novel Model to Stratify Risk of Recurrence. Cancers.

[14] Mayer P., Kraft A., Witzel H.R., Marnet N., Hörner N., Roth W., Heinrich S., Hackert T., Bergmann F., Kauczor H.-U. (2021). Restricted Water Diffusion in Diffusion-Weighted Magnetic Resonance Imaging in Pancreatic Cancer is Associated with Tumor Hypoxia. Cancers.

[15] Puglisi C., Giuffrida R., Borzì G., Di Mattia P., Costa A., Colarossi C., Deiana E., Picardo M.C., Colarossi L., Mare M. (2020). Radiosensitivity of Cancer Stem Cells Has Potential Predictive Value for Individual Responses to Radiotherapy in Locally Advanced Rectal Cancer. Cancers.

[16] Paydary K., Reizine N., Catenacci D.V.T. (2021). Immune-Checkpoint Inhibition in the Treatment of Gastro-Esophageal Cancer: A Closer Look at the Emerging Evidence. Cancers.

[17] Semeraro G.C., Cipolla C.M., Cardinale D.M. (2021). Role of Cardiac Biomarkers in Cancer Patients. Cancers.

[18] Honrubia-Peris B., Garde-Noguera J., García-Sánchez J., Piera-Molons N., Llombart-Cussac A., Fernández-Murga M.L. (2021). Soluble Biomarkers with Prognostic and Predictive Value in Advanced Non-Small Cell Lung Cancer Treated with Immunotherapy. Cancers.

[19] Broggi G., Musumeci G., Puzzo L., Russo A., Reibaldi M., Ragusa M., Longo A., Caltabiano R. (2019). Immunohistochemical Expression of ABCB5 as a Potential Prognostic Factor in Uveal Melanoma. Appl. Sci..

[20] Foti P.V., Travali M., Farina R., Palmucci S., Spatola C., Liardo R.L.E., Milazzotto R., Raffaele L., Salamone V., Caltabiano R. (2021). Diagnostic methods and therapeutic options of uveal melanoma with emphasis on MR imaging-Part II: Treatment indications and complications. Insights Imaging.

[21] Gajdzis M., Kaczmarek R., Gajdzis P. (2021). Novel Prognostic Immunohistochemical Markers in Uveal Melanoma-Literature Review. Cancers.

[22] Beyoğlu D., Idle J.R. (2021). Metabolic Rewiring and the Characterization of Oncometabolites. Cancers.

[23] González-Gascón-y-Marín I., Muñoz-Novas C., Rodríguez-Vicente A.-E., Quijada-Álamo M., Hernández-Sánchez M., Pérez-Carretero C., Ramos-Ascanio V., Hernández-Rivas J.-Á. (2021). From Biomarkers to Models in the Changing Landscape of Chronic Lymphocytic Leukemia: Evolve or Become Extinct. Cancers.

[24] Torrisi F., Vicario N., Spitale F.M., Cammarata F.P., Minafra L., Salvatorelli L., Russo G., Cuttone G., Valable S., Gulino R. (2020). The Role of Hypoxia and SRC Tyrosine Kinase in Glioblastoma Invasiveness and Radioresistance. Cancers.

[25] Russo G.I., Sholklapper T.N., Cocci A., Broggi G., Caltabiano R., Smith A.B., Lotan Y., Morgia G., Kamat A.M., Witjes J.A. (2021). Performance of Narrow Band Imaging (NBI) and Photodynamic Diagnosis (PDD) Fluorescence Imaging Compared to White Light Cystoscopy (WLC) in Detecting Non-Muscle Invasive Bladder Cancer: A Systematic Review and Lesion-Level Diagnostic Meta-Analysis. Cancers.

[26] Diaz-delCastillo M., Andrews R.E., Mandal A., Andersen T.L., Chantry A.D., Heegaard A.-M. (2021). Bone Pain in Multiple Myeloma (BPMM)—A Protocol for a Prospective, Longitudinal, Observational Study. Cancers.

